# Effect of spineless cactus intake (*Opuntia ficus-indica*) on blood glucose levels in lactating sows and its impact on feed intake, body weight loss, and weaning-estrus interval

**DOI:** 10.1007/s11250-017-1295-7

**Published:** 2017-04-28

**Authors:** Gerardo Ordaz-Ochoa, Aureliano Juárez-Caratachea, Rosa Elena Pérez-Sánchez, Rafael María Román-Bravo, Ruy Ortiz-Rodríguez

**Affiliations:** 1 Instituto de Investigaciones Agropecuarias y Forestales, Universidad Michoacana de San Nicolás de Hidalgo, Km 9.5 Carretera Morelia-Zinapécuaro, Tarímbaro, Michoacán Mexico; 2 Facultad de Agrobiología “Presidente Juárez”, Universidad Michoacana de San Nicolás de Hidalgo, Paseo Gral. Lázaro Cárdenas y Berlín S/N Col Viveros, C.P. 60170 Uruapan, Michoacán Mexico; 3 Facultad de Ciencias Veterinarias, Universidad del Zulia, Av. 25, Sect. Manzana de Oro, Núcleo Grano de Oro, Maracaibo, Venezuela; 4 Facultad de Medicina Veterinaria y Zootecnia, Universidad Michoacana de San Nicolás de Hidalgo, Av. Acueducto S/N esquina Tzintzuntzan, Col Matamoros, C.P. 58130 Morelia, Michoacán Mexico

**Keywords:** Feeding, Hypophagia, Lactation, Glycaemia

## Abstract

The effect of spineless cactus intake (*Opuntia ficus-indica*) on blood glucose (BG) levels in lactating sows and its impact on daily and total feed intake (dFI^−1^ and TFI, respectively), body weight loss (BWL), and weaning-estrus interval length (WEI) were evaluated. Thirty-four hybrid (Yorkshire × Landrace × Pietrain) sows in lactation phase were used. Sows were divided into two groups: G1 (*n* = 17) where they received commercial feed and G2 (*n* = 17) provided with commercial feed plus an average of 2.0 ± 0.5 kg spineless cactus, based on a sow’s body weight. The variables evaluated were BG, dFI^−1^, TFI, BWL, and WEI. Statistical analysis was performed by using a fixed and mixed model methodology, under a repeated measurements experiment. Group effects were found on all analyzed variables (*P* < 0.05). The BG was lower in G2 (55.2 and 64.5 mg/dL pre- and post-prandial, respectively), compared to that in G1 (70.9 and 80.1 mg/dL pre- and post-prandial, respectively) (*P* < 0.05). G2 showed better performance than G1 for dFI^−1^, BWL, and WEI (*P* < 0.05) whose averages were 5.5 ± 1.8 kg, 7.4 ± 4.5%, and 5.3 ± 1.2 days, respectively. Averages for these variables in G1 were 4.7 ± 1.5 kg, 16.8 ± 4.6%, and 6.1 ± 1.6 days, respectively. Intake of spineless cactus reduced BG levels in lactating sows, generating greater dFI^−1^, lower BWL at the end of lactation, and a lower WEI.

## Introduction

The feeding of the sow during lactation is among the most important indicators to maintain the productivity of the swine production systems (Segura et al., 2013; Xie et al., 2015). During this stage of production, the sows experience lactational physiologic hypophagia, which is associated with a gradual development of insulin resistance (Koketzu et al., 1998; Schenkel et al., 2010), and therefore, to an increase in blood glucose (Pére and Etienne, 2007; Klosterbuer et al., 2012). The increase of blood glucose in lactating sows causes alterations in appetite, reflected in a decrease in voluntary feed intake during the first week post-farrowing (Rigón et al., 2008), which is manifested in body weight loss of the sows and in the decrease of reproductive and productive post-lactation indicators (weaning) (Schenkel et al., 2010).

A sows’ body weight loss caused by the lactational physiologic hypophagia is not only associated with the reduced voluntary feed intake but is also the result of energy imbalance and the removal of corporal reserves during lactation (Moreira et al., 2013). However, only if the loss of body weight is greater than 10% at the end of lactation, the reproductive processes subsequent to weaning are negatively affected (Cools et al., 2014); it mainly generates a delayed response in the resumption of ovarian activity and increases the weaning-estrus interval, reducing the fertility and prolificacy of the sows (Schenkel et al., 2010).

Given the above reasoning, nutritional alternatives for lactating sows are required to solve or minimize the effects of the lactational physiologic hypophagia and at the same time preserve animal health (Quesnel et al. 2009), production performance, and product quality (Tikabo et al., 2006). Thus, the forage spineless cactus (*Opuntia* spp), for its hypoglycemic quality, high fiber content, and digestible energy (Alarcon et al., 2003; Pinos et al., 2010), can be a nonconventional alternative in the nutritional strategy of lactating sows, to counter the lactational physiologic hypophagia effect and to improve reproductive and productive activity after weaning. Therefore, the objective of this research was to evaluate the effect of spineless cactus intake (*Opuntia ficus-indica*) on blood glucose levels in lactating sows and its impact on daily and total feed intake, body weight loss, and weaning-estrus interval.

## Materials and methods

This research was carried out at the Swine Unit of “La Posta Zootécnica” belonging to the Veterinary Medicine and Husbandry Faculty of Universidad Michoacana de San Nicolás de Hidalgo (FMVZ-UMSNH), Tarímbaro, Michoacán, México, located at km 9.5 Morelia-Zinapécuaro Road; 19° 46′ N, 101° 08′ W, and altitude of 1855 m (INEGI, 2010).

### Animals, diets, and housing

Thirty-four hybrid sows (Yorkshire × Landrace × Pietrain), with 2.6 ± 1.5 average farrowing (1–7 farrowing range), were selected at random from the reproductive herd on the evaluated swine unit. The sows were served by natural mating with hybrid boars (Yorkshire × Pietrain) when presenting post-weaning estrus and were housed by groups (*n* = 7) in 16-m^2^ pens during 108 days of gestation. All sows were fed 2.0 kg day^−1^ of commercial feed during the first two thirds of gestation (89.9% dry matter, 12.5% crude protein, 3.7% crude fat, 3.1% crude fiber, 10% crude ash, all on as-fed basis); of the last third, until the 108th day of gestation, sows were fed 2.5 kg day^−1^ divided in two rations: 08:00 and 14:00 h. The feed was provided in individual concrete feeders. The diet composition is shown in Table 1. The water supply was ad libitum through an automatic nipple drinker.

**Table 1:** Ingredients and nutrient composition of conventional gestation diet, conventional lactation diet (group 1), and experimental lactation diet (group 2)

Item	Gestation diet	Lactation diet	
Ingredient, g/kg		Group 1	Group 2
Sorghum	824.0	649.7	649.7
Soybean paste	60.0	100.0	100.0
Canola paste	61.5	185.3	185.3
Orthophosphate	11.8	5.4	5.4
Calcium carbonate	14.0	12.4	12.4
Soy oil	22.0	38.5	38.5
Lysine	1.2	2.5	2.5
Salt	4.0	4.0	4.0
Vitamin and mineral premix^a^	2.0	2.5	2.5
Nutrient composition for spineless cactus (*O. ficus-indica*)^b^			
Crude protein, %			5.6
Crude fat, %			0.2
Fiber, %			28.8
Humidity, %			88.6
Ash, %			24.5
Nitrogen-free elements, %			40.8
Mucilage, g 300 g^−1^ dry base			2.6
Nutrient composition^c^			
Metabolizable energy, Mcal/kg^d^	2.3	2.3	2.3
Crude protein, %	12.5	17.5	17.3
Crude fat, %	3.7	4.5	4.4
Fiber, %	3.1	4.3	4.7
Humidity, %	12.0	12.0	13.8
Ash, %	10.0	10.0	12.9
Calcium, %^d^	0.75	0.75	0.76
Phosphorus, %^d^	0.60	0.60	0.59
Lysine, %^d^	0.52	0.95	0.94
Met-Cist, %^d^	0.43	0.59	0.59

^a^Provided per kilogram of diet: Cu 30 mg; Fe 160 mg; Zn 160 mg; Mn 55 mg; Se 0.5; Cr 0.2 mg; vitamin A 14,200 IU; vitamin D_3_ 2800 IU; vitamin E 125 mg; vitamin K_3_ 5 mg; vitamin B_1_ 2.4 mg; vitamin B_2_ 8.7 mg; vitamin B_6_ 4.5 mg; vitamin B_12_ 0.05 mg; pantothenic acid 35 mg; folic acid 6 mg

^b^ Supply of spineless cactus in fresh base was given once a day in the morning throughout the lactation phase. Quantity was 1% of the sow’s pre-parity body weight

^c^ In order to determine the nutritional composition of the diet supplemented with spineless cactus, 1% of spineless cactus was added in dry base to the conventional feed sample before bromatological analysis

^d^ Chemical concentrations calculated using feed ingredient values from NRC (1998)

One week before the probable farrowing date (day 109 of gestation), the sows were randomly selected to form each one of the two groups (G) established in the experimental design: G1 (*n* = 17), which was offered commercial feed, and G2 (*n* = 17), sows with commercial feed plus spineless cactus. The sows were then transferred to the farrowing house where they were fed a lactation diet until the farrowing day (the ingredients and nutritional composition are shown in Table 1). After farrowing, all sows in both groups were fed ad libitum during the 21 days of lactation. The only difference in the feeding of the post-farrowing sows was the addition of the spineless cactus (*O. ficus-indica*), as-fed basis (AF), to the diet of G2 (Table 1): commercial feed +1% of spineless cactus (according to the weight of the pre-partum sow). The corresponding spineless cactus quantity, added to the sow^−1^ diet, was on average 2.0 ± 0.5 kg spineless cactus. Due to the variety of the spineless cactus (no spikes), the cladodes were only fragmented into 3 × 2 cm pieces approximately and added to the corresponding diet at 08:00 h.

The sows in both groups were monitored and had the same husbandry practices during lactation, which was done in the farrowing house (maternity). This area has an installed capacity for six cages for farrowing and lactation; each cage has a bucket-type stainless steel feeder (44.5 cm/wide, 37.0 cm/height, and 33.0 cm/deep) and an automatic nipple drinker. The temperature in the maternity area remained constant (18 °C) during the experimental period (temperature for the sows and their litters); an automatic infrared heater Holme® brand with power from 750 to 1500 W, which was regulated at 18 °C. The ventilation was through curtains.

### Experimental procedures

The variables evaluated sow^−1^ group^−1^ were blood glucose (BG), daily and total feed intake (dFI^−1^ and TFI, respectively), body weight loss (BWL), and weaning estrus-interval (WEI). BG was determined by a glucometer for human use (ACCU-CHEK Performan®), according to the methodology described by Perez et al. (2015b). Blood samples were taken at 8:00 h by right ear vein puncture on gestation days 85, 100, and 110 and lactation days 3, 7, 10, 14, 17, and 21, prior to a fasting period of 8 h. Feed intake was determined with a portable digital scale (model CRS5000, TOR REY®, Mexico) with capacity of 0.100 to 500 kg. Rejected feed sow^−1^ group^−1^ was weighed daily in the morning, prior to feeding, with the aim of determining the dFI^−1^ from the day before and TFI. The live weight of the sows when entering the farrowing house (7 days pre-farrowing) and at weaning (21 days post-farrowing) was obtained using a fixed electronic scale (STG-1500-T1500SL, OCONY®, México) with capacity from 1 to 1500 kg. The BWL (kg and %) was determined at the end of lactation through the following formulas:

$$ {\mathrm{BWL}}_{\mathrm{kg}}=\mathrm{Weight}\ \mathrm{of}\ \mathrm{the}\ \mathrm{sow}\ \mathrm{pre}\ \mathrm{farrowing} - \mathrm{Weight}\ \mathrm{of}\ \mathrm{the}\ \mathrm{sow}\ \mathrm{at}\ \mathrm{weaning} $$

$$ {\mathrm{BWL}}_{\%}=100 - \left(\frac{\mathrm{Weight}\ \mathrm{of}\ \mathrm{the}\ \mathrm{sow}\ \mathrm{at}\ \mathrm{weaning} \times 100}{\mathrm{Weight}\ \mathrm{of}\ \mathrm{the}\ \mathrm{sow}\ \mathrm{pre}\ \mathrm{farrowing}}\right) $$.

### Statistical analysis

The data was analyzed by ANOVA using the methodology of the fixed and mixed effects models (SAS, 2010). Data for sows BG and dFI^−1^ were analyzed using ANOVA for repeated measures experiment, with sow nested within group as a random effect and group, day, and group × day interaction, and the adjustment for the linear effect of the body weight pre-farrowing as fixed effects. The model for TFI, LBW, and WEI included group as a fixed effect and taking pre-farrowing sow body weight and litter size at weaning as covariates. Differences between means were estimated by using the least square means SAS statement setting *α* = 0.05. Thus, the explicitly models used were:

$$ {Y}_{i jkl}=\mu +{G}_i+{S(G)}_{j(i)}+{D}_k+{G\times D}_{i k}+{\beta}_1{X}_{1 j}+{\upvarepsilon}_{i jkl} $$

Where:

*Y*_*ijkl*_ = response variable: BG and dFI^−1^; *μ* = constant common to the population; *G*_*i*_ = fixed effect of *i* ^−^th group, with *i* = 1, 2; *S*(*G*)_*j*(*i*)_ = random effect of the *j* ^−^th sow, nested within the *i* ^−^th group, with *j =* 1, 2,…, 17 and *i* = 1, 2; *D*_*k*_ = fixed effects of the *k* ^−^th lactation day, with *k* = 1, 2, 3,…, 21; *G*×*D*_*ik*_ = fixed effects of interaction of *i* ^−^th group in *k* ^−^th lactation day; *X*_1*j*_ = effect of covariate pre-farrowing body weight of *j-*sow; *ε*_*ijkl*_ = random effect associated with each observation (∼NID = 0, *σ* ^2^_e_).

$$ {Y}_{i j}=\mu +{G}_i+{\beta}_1{X}_{1 j}+{\beta}_2{X}_{2 j}+{\upvarepsilon}_{i j} $$

Where:

*Y*_*ij*_ = response variable: TFI, BWL, and WEI; *μ* = constant common to the population; *G*_*i*_ = fixed effects of the *i* ^−^th group, with *i* = 1, 2; *X*_1*j*_ = effect of the covariate pre-parity body weight for *j*-sow; *X*_2*j*_ = effect of the covariate litter weight at weaning for *j-*sow; *ε*_*ij*_ = random effect associated with each observation (∼NID = 0, *σ* ^2^_e_).

## Results

There was no group effect on BG levels pre- and post-prandial during the last third of gestation (*P* = 0.085): 70.3 ± 7.2 vs 75.2 ± 7.9 mg/dL for the control group (G1) and experimental group (G2), respectively. The post-prandial BG levels were 79.8 ± 8.2 mg/dL for G1 and 83.2 ± 6.7 mg/dL for G2. However, during lactation, there were group effects on BG levels pre- and post-prandial (*P* < 0.001). Pre-prandial BG levels in G1 were greater during lactation (*P* < 0.05): 70.9 ± 8.2 mg/dL in comparison with 55.2 ± 8.5 mg/dL from G2. The post-prandial BG levels showed the same effect; it was greater in G1 than in G2 (*P* < 0.05; Table 2).

**Table 2:** Least squares mean for blood glucose levels pre- and post-prandial according to lactation interaction group × day

Day	Control group (G1)		Group fed spineless cactus (G2)		Contrast *P* value*	
	BG pre-pandrial	BG post-pandrial	BG pre-pandrial	BG post-pandrial	1	2
85 to 110^&^	70.3^1^ ± 7.2	79.8^1^ ± 8.2	75.2^1^ ± 7.9	83.2^1^ ± 6.7	<0.0001	<0.0001
1 to 7^†^	72.0 ^a1^ ± 8.3	81.5^a1^ ± 9.0	59.7^a2^ ± 5.4	67.2^a2^ ± 12.1	<0.0001	<0.0001
8 to 14^†^	71.1^a1^ ± 8.5	81.4^a1^ ± 10.6	46.4^a2^ ± 7.5	58.7^b2^ ± 5.7	<0.0001	<0.0001
15 to 21^†^	69.2^a1^ ± 7.7	76.9^a1^ ± 12.9	57.1^a2^ ± 7.1	66.1^a2^ ± 9.5	<0.0001	<0.0001

^&^Gestation phase

^**†**^ Lactation phase

^a,b^Different letters indicate statistical difference (*P* < 0.05) within the column

^1,2^Different numerals indicate statistical difference (*P* < 0.05) between groups for BG pre-prandial and post-prandial, respectively

*Contrast: 1, BG pre-prandial group 1 vs BG pre-prandial group 2; 2, BG post-prandial group 1 vs BG post-prandial group 2

**Fig. 1:**
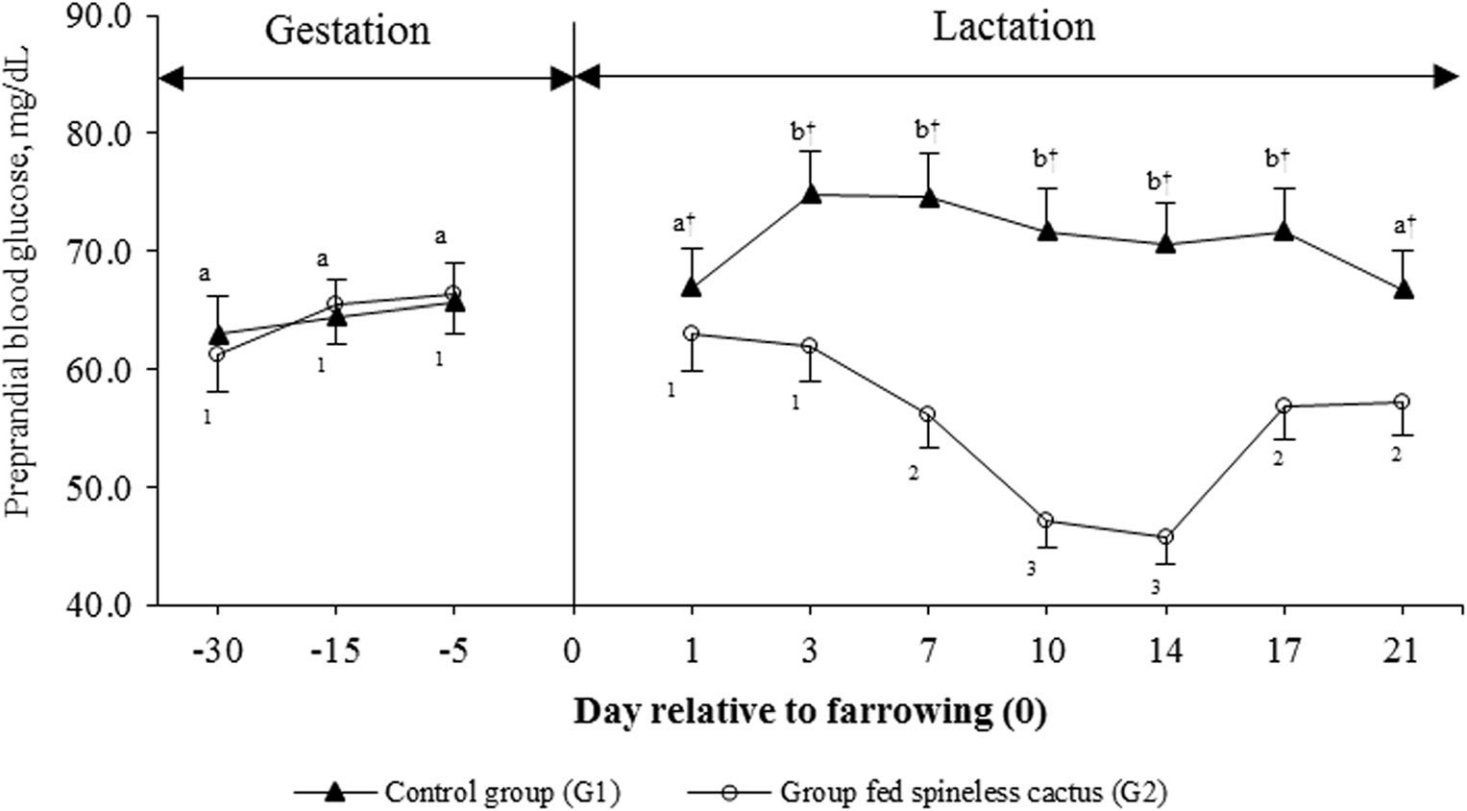
Least squares means for pre-prandial blood glucose levels (mean ± SD) according to the lactation day in sows fed a conventional diet (*G1*, *n* = 17) and sows fed a conventional diet supplemented with spineless cactus (*G2*, *n* = 17). *Different letters* indicate statistical difference (*P* < 0.05) between day of lactation for *G1*. *Different numerals* indicate statistical difference (*P* < 0.05) between days of lactation for *G2*. ^†^Statistical difference (*P* < 0.05) between groups

BG level reduction in G2 sows did not show immediately; interaction group × day was highly significant (*P* < 0.001). At 24-h post-administration of the spineless cactus, a 7.9% reduction of the pre-prandial BG was observed with respect to the pre-prandial BG levels of the sows in G1 (66.4 mg/dL); at 168 h, the reduction was 24.7%, and at 336 h, the greatest reduction of pre-prandial BG was observed (35.3%) in comparison to that of G1 (Fig. 1). The analysis by orthogonal polynomials indicated that the response in both groups can be described by a second-degree polynomial, and the estimated prediction equations for each group were G1 ${\widehat{Y}}_i=68.5152+1.0139 X-0.0527{X}^2$ and G2 ${\widehat{Y}}_i=66.5247-2.7426 X+0.1118{X}^2$. Deriving both equations and equaling to 0, the critical points for each group were 9.6 for G1 and 12.2 for G2; this implies that the sows in G1 show the greatest level of pre-prandial BG around day 9.6 and the sows in G2 show the lowest level of pre-prandial BG around day 12.2.

**Fig. 2:**
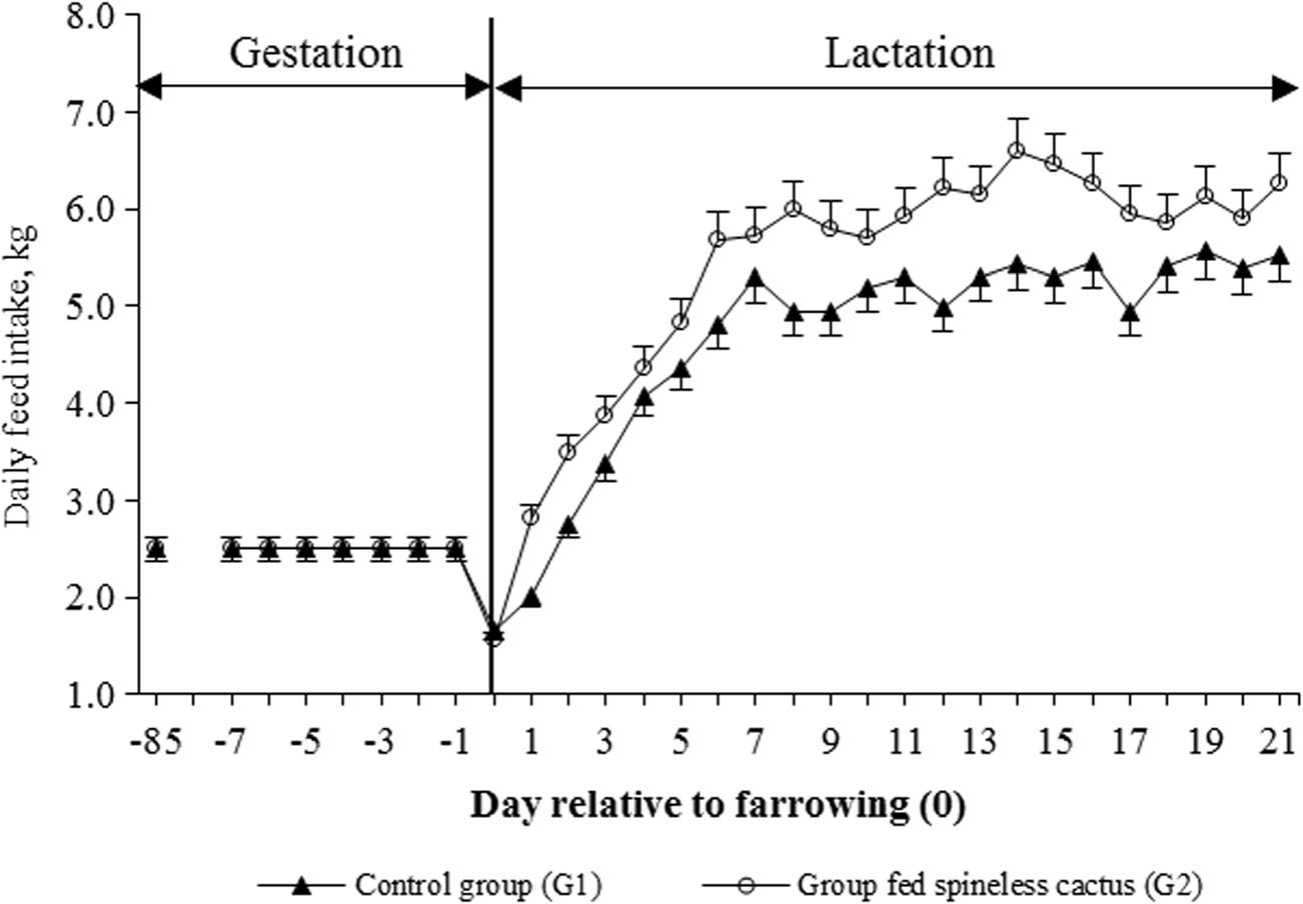
Least squares means for voluntary feed intake (mean ± SD) during the last third of gestation (30 days pre-farrowing) and lactation (21 days post-farrowing) in sows fed with conventional diet (*G1*, *n* = 17) and sows fed a diet supplemented with spineless cactus (*G2*, *n* = 17)

There was no group effect regarding the dFI^−1^ per sow during the last third of gestation (*P* = 0.090). During lactation, the feed intake increased (*P* < 0.05) in sows of G2 (4.4 ± 1.7 kg day^−1^) in the first week post-farrowing in comparison with the dFI^−1^ from the sows of G1 (Table 3). The average dFI^−1^ during the 21 days of lactation were 4.7 ± 1.5 and 5.5 ± 1.8 kg for sows in G1 and G2, respectively (*P* < 0.05). Regarding the highest dFI^−1^ for each group, their averages were 5.2 and 6.6 kg for G1 and G2 groups, respectively (Fig. 2). Moreover, increase of dFI^−1^ was not linear during the 21-day lactation period, since there was a decline in voluntary feed intake at the beginning of the third lactation week (Fig. 2).

**Table 3:** Least squares means for average feed intake day^−1^ for lactating sows according to group

	Control group (G1)	Group fed spineless cactus (G2)					*P* value*
	CF	CF	CF + SC _*FB*_	CF + SC _*DB*_	SC _*FB*_	SC _*DB*_	
Day 85 to 110^&^	2.5^a^	2.5^a^	–	–	–	–	0.087
Day 1 to 7^†^	3.8 ^a1^ ± 1.5	4.4 ^b1^ ± 1.7	6.2^1^ ± 2.2	4.6^1^ ± 1.8	1.7^1^ ± 0.6	0.20^1^ ± 0.06	<0.001
Day 8 to 14^†^	5.1 ^a2^ ± 1.3	5.9 ^b2^ ± 1.9	7.5^2^ ± 2.1	6.1^2^ ± 1.9	1.9^1^ ± 0.11	0.19^1^ ± 0.07	<0.001
Day 15 to 21^†^	5.4 ^a2^ ± 1.4	6.2 ^b2^ ± 1.6	7.7^2^ ± 1.6	6.3^2^ ± 1.6	1.5^1^ ± 0.7	0.17^1^ ± 0.10	<0.001
General average	4.7 ^a^ ± 1.5	5.5 ^b^ ± 1.8	7.1 ± 1.9	5.7 ± 1.8	1.6 ± 0.6	0.19 ± 0.09	<0.001

*CF* commercial fed, *SC* spineless cactus, *BH* fresh base, *BS* dry base

^&^Gestation phase

^†^ Lactation phase

^a,b^Different letters indicate statistical differences (*P* < 0.05) for the intake CF/group

^1,2^Different numerals indicate statistical differences (*P* < 0.05) within the column

*Probability for the group effect on CF only

**Fig. 3:**
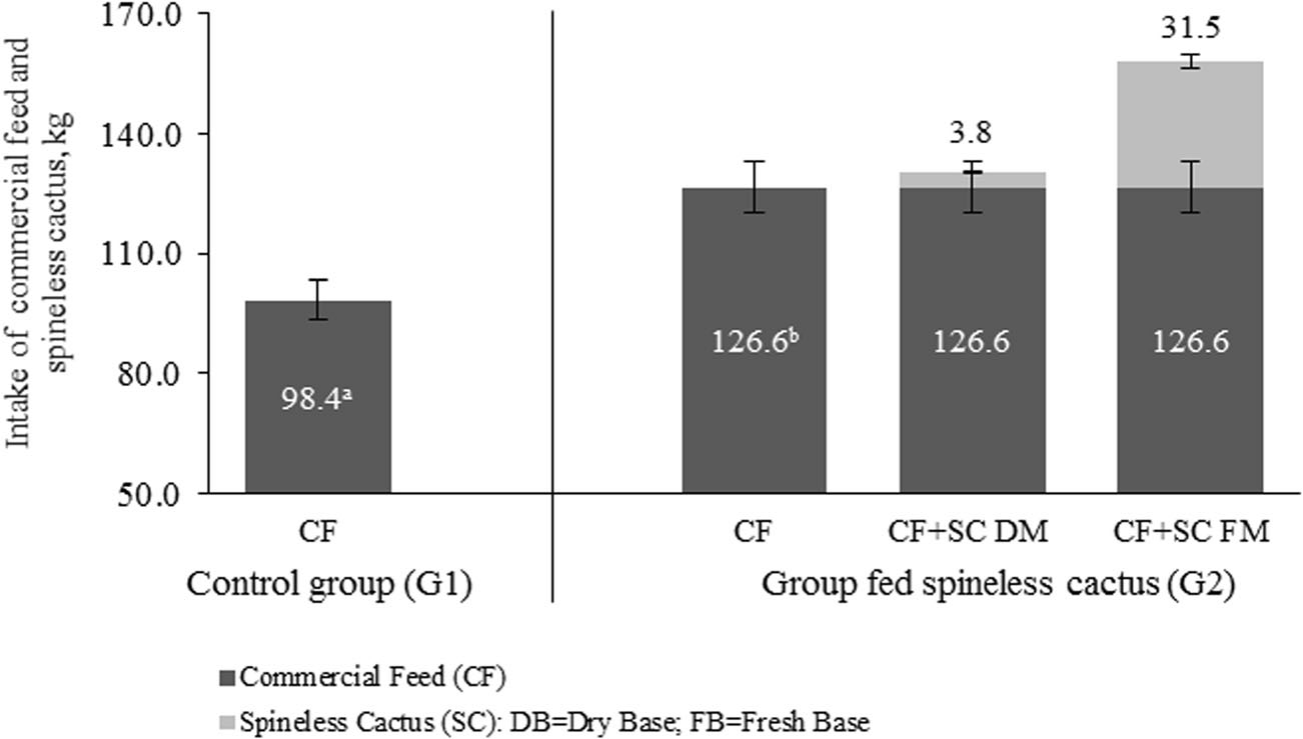
Least squares means for commercial feed (*CF*) total intake by sows fed conventionally (*G1*, *n* = 17) and total intake of commercial feed plus spineless cactus (*CF + SC*), in dry and fresh base (*DM* and *FM*, respectively) for sows fed a commercial diet plus spineless cactus (*G2*, *n* = 17), during lactation phase (21 days). Mean ± SD. *Different letters* indicate statistical difference (*P* < 0.05) between groups regarding *CF* total intake

The increase of dFI^−1^ sow^−1^ week^−1^ observed in G2 impacted TFI during the whole lactation phase, with a total of 126.6 kg of commercial feed in 21 days, resulting in 28.2 kg more of commercial feed compared to sows of G1 (Fig. 3). Moreover, G2 sows had 31.5 ± 7.4 kg of spineless cactus in fresh base in addition to the commercial feed consumed during the 21-day lactation phase, resulting in a TFI 158.1 kg sow^−1^.

**Fig. 4:**
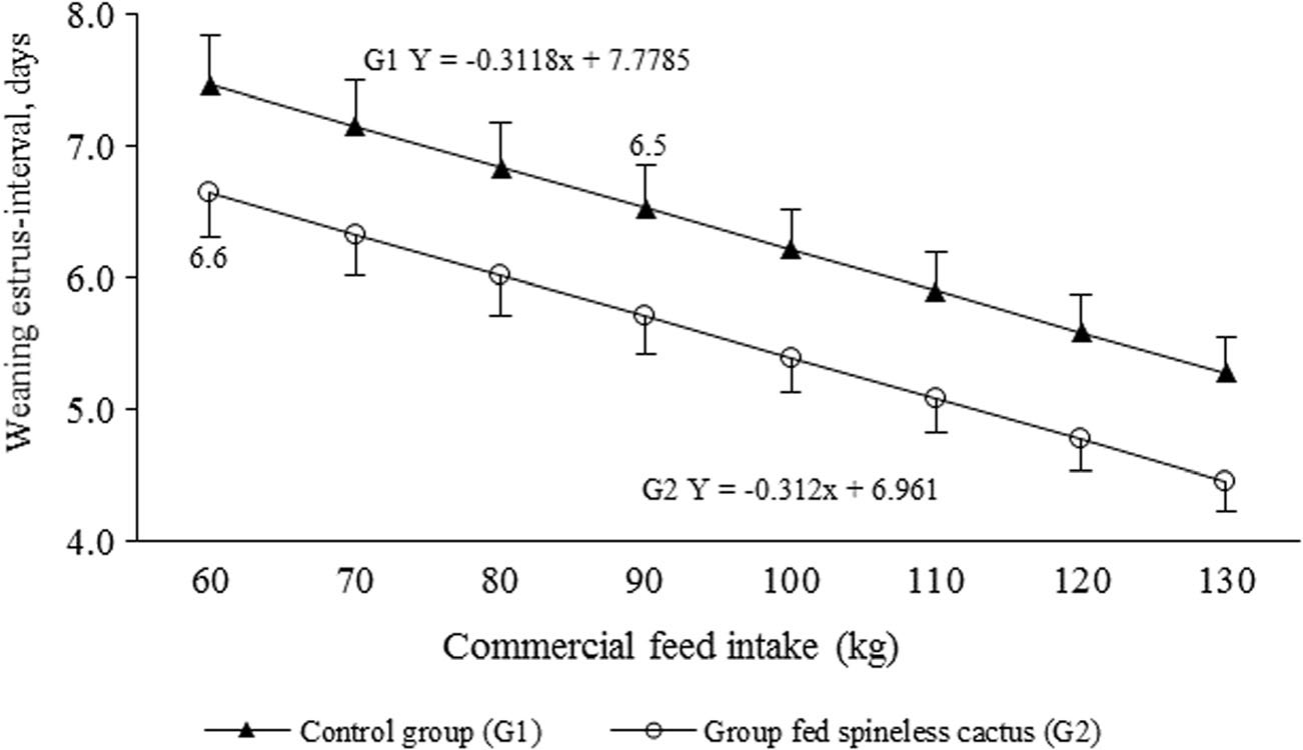
Commercial feed intake (kg) linear effect during lactation phase (21 days) on weaning estrus-interval (mean ± SD) in sows fed conventionally (*G1*, *n* = 17) and sows whose diet was supplemented with spineless cactus (*G2*, *n* = 17)

The sows that consumed spineless cactus during lactation (G2) showed a lower percentage of BWL in comparison to sows of G1 (*P* < 0.05; Table 4). It could also be established that the sows with a lower BWL (G2) showed a lower WEI compared to those in G1 (*P* < 0.05): 5.3 ± 1.2 and 6.1 ± 1.6 days, respectively. Finally, the association between TFI and WEI was *r* = 0.36; (*P* < 0.001), and that of spineless cactus intake and WEI was *r* = −0.40; (*P* < 0.001). And the regression estimator of spineless cactus intake on TFI was *β*_1_ = 1.1 kg; (*P* < 0.05); whereas that of spineless cactus intake on WEI was *β*_1_ = −0.03 days; (*P* < 0.05), and the linear regression of TFI on WEI was *β*_1_ = −0.06 days; (*P* < 0.05). It can be inferred that for each kilogram of spineless cactus intake during lactation, the TFI increased in 1.1 kg, which originates in a reduction of the WEI in 0.3 days for each kilogram of feed consumed and of 0.6 days per each kilogram of spineless cactus intake. Therefore, the sows that consumed spineless cactus not only showed a lower WEI (*P* < 0.05), but also the optimal WEI is achieved (7 days) with less TFI due to the extra energy intake provided by the spineless cactus (Fig. 4).

**Table 4:** Least squares means for sows’ body weight loss during lactation phase and weaning estrus-interval according to the group

	Control group (G1)	Group fed spineless cactus (G2)	*P* value
Body weight loss, kg	25.7^a^ ± 11.3	16.8^b^ ± 10.0	<0.0001
Body weight loss, %	16.8^a^ ± 4.6	7.4^b^ ± 4.5	<0.0001
Weaning-estrus interval, days	6.1^a^ ± 1.6	5.3^b^ ± 1.2	<0.0001

^a,b^Different letters indicate statistical difference (*P* < 0.05) within the row

## Discussion

The reduced BG levels in lactating sows fed the diet supplemented with spineless cactus (Table 2) is consistent with the effects observed in humans and other animal species after consuming spineless cactus (Alarcón et al., 2003; Halmi et al., 2013). In rabbits, it has been documented that spineless cactus intake reduces glucose levels up to 21.2% (*P* < 0.05) (Halmi et al., 2013); apparently, the hypoglycemic effect of the cactus is due to the dietary fiber, especially pectin, which causes a decrease in carbohydrate absorption by the pectin-gel formation (Sánchez et al., 2008). In addition, diets high in dietary fiber increase microbial enzymatic activity, affecting the activity of α-glucosidase and β-galactosidase, resulting in the inhibition of the glycosidic bond hydrolysis (Igho et al., 2015). Nevertheless, maximum reduction (35.3%) in pre-prandial BG levels in G2 sows was not observed until 336-h post-initiation of the treatment with spineless cactus (Fig. 1); these results show the possibility of modifying the lactational physiological hypophagia effect and increased feed intake in lactating sows by decreasing BG levels within the first 12 days of post-parity through the addition of spineless cactus to the diet.

A decrease in BG levels, due to spineless cactus intake, may be a consequence of an increase in insulin sensitivity due to the suppression of hepatic glucose production (Andrade y Wiedenfeld, 2011). Possibly, the nonfermentable digestible dietary fiber of the spineless cactus increases the release of intestinal peptides similar to glucagon-1 (GLP-1) (Chen et al., 2014), causing an increase of insulin production, inhibiting the release of glucagon, and influencing the renewal of intestinal epithelial cells (Jasleen et al., 2002). Additionally, the high calcium content (0.59 mg/100 g) of the cactus (Pinos et al., 2010) could stimulate insulin secretion by closing the K^+^/ATP channels and depolymerize the membrane because of the increase of Ca^2+^ in the plasma membrane of the cells: the main key to insulin release (Pari y Latha, 2005).

The metabolic route of the lactational physiologic hypophagia is associated with the increase of the hepatic gluconeogenesis and the increase, from lipolysis, in the formation of nonsteroidal fatty acid precursors of leptin (Paspala et al., 2012). However, the increase of leptin post-parity originates hypophagia due to the following: (1) blocking of the ghrelin receptor at the gastric level and (2) blocking the orexigenic action of the neuropeptides-*Y* and agouti protein type at the hypothalamic level (Ylonem et al., 2003), whose effect is more evident during the first week of post-parity (Pére y Etienne, 2007; Segura et al., 2013), which is consistent with the results found in this research (Table 3).

In addition to the hypoglycemic effect of the spineless cactus, its high fiber content (300 g/kg) (Pinos et al., 2010) could have possibly caused (i) the capturing of cholesterol and triglycerides (appetite regulators) and (ii) greater gastric distension on the sows, which allowed greater daily feed intake (Fig. 2) and increased (*P* < 0.05) TFI during lactation (Fig. 3).

On the contrary, in G1, the absence of a factor that decreases the BG levels, cholesterol, low-density lipoproteins, and triglycerides in the blood during lactation maintained active metabolic mechanisms that cause lactational hypophagia (Paspala et al., 2012) and its negative effects on voluntary feed intake (Rigón et al., 2008). The fact that the increase in dFI^−1^ of sows in G2 was not lineal during the 3 weeks of lactation (Fig. 2) is that on the 15th day of lactation, post-farrowing ovarian activity initiates, which is characterized by the increase of reproductive hormones: follicle stimulating hormone (FSH), luteinizing hormone (LH), and estrogens (E2); these hormones reduce dFI^−1^ by blocking the chemical mediators (neuropeptides-*Y*) with orexigenic effect at the hypothalamic level (Pére y Etienne, 2007; Barb et al., 2008). Thus, the decrease in dFI^−1^ at the beginning of the third week of lactation can be considered physiologically as normal.

The dFI^−1^ of the lactating sow has a direct repercussion on the TFI at the end of lactation. However, there are differences regarding the TFI of the sows during lactation, since previous researches report a TFI ranging between 92.8 (Pérez et al., 2015a) and 103.3 kg (Cools et al., 2014) of feed in 21-day lactations. This variable (TFI) is affected primarily by the sows’ genotype, dFI^−1^ during gestation, corporal condition at farrowing, feeding frequency, environmental temperature, water availability, age, and metabolic physiology of the sow during lactation (Pére y Etienne, 2007; Olsson et al., 2011).

The total spineless cactus intake from the sows in G2 is an important aspect to consider (31.5 ± 7.4 kg AF) and can establish the gastric capacity during lactation (Table 3). In addition, this spineless cactus intake (1.6 ± 0.5 kg day^−1^ sow^−1^) was able to counteract the negative effects of lactational hypophagia by stimulating a greater feed intake (12.4%) and decreasing the BWL in sows of G2 during lactation (Table 4).

It is possible that the spineless cactus, besides its hypoglycemic effects, can improve the digestive processes of lactating sows, since it has been observed that the greater live weight gain in animals fed diets complemented with cactus is due to the high soluble carbohydrate content (53.9%) (Tikabo et al., 2006). Additionally, the nonstarch-soluble polysaccharides, present in the cactus, increase the viscosity of the alimentary bolus (Chen et al., 2014) and inhibit the phosphodiesterase in the intestinal smooth muscle (Baldassano et al., 2010), causing the reduction of gastric emptiness and the transit rate of the nutritional content through the gastrointestinal tract (Wang et al., 2003), generating greater nutrient absorption (Le Goff et al., 2002). Also the nonstarch polysaccharides, when undergoing fermentation by the microbiota of the colon, lead to a greater production of volatile fatty acids, including acetate, propionate, butyrate, and valeric acid (Cani et al., 2006), metabolites intended to supply energy to the organism (Chen et al., 2014). This longer time of the feed in the gastrointestinal tract, triggered by the spineless cactus intake, could generate lower BWL of the sows during lactation (Table 4). The incorporation of fermentable nonstarch polysaccharides modifies the activity of the intestinal microbiota and makes digestibility more efficient, improving with the weight and age of the swine (Rajesh y Berrocoso, 2016).

In this regard, it has been determined that a BWL greater than 10% during lactation affects subsequent productive indicators, like the increase in the WEI, repeat-service percentage, and litter size reduction in the next farrowing (Cools et al., 2014). This agrees with the results from this research, especially of the WEI (Table 4), where the sows with lower BWL (G2) showed lower WEI. The reduction of the WEI of the sows from G2 was possibly due to the increased carbohydrates in the diet provided by the spineless cactus; as it has been observed that the implementation of glycogenic treatments trigger estrus (Viñoles et al., 2008); and combined with the energy content of the cactus (13.0 MJ/kg) (Nefzaoui y Ben Salem, 2002), the fiber content and its hypoglycemic effect could have positively affected the follicular dynamic of the ovaries (Sakly et al., 2013).

In the reproductive processes of the sows, the energy balance during lactation is essential for the resumption of reproductive activity post-weaning and the insulin appears to be the main modulator post-partum between the nutritional balance and the energy removed for ovarian reactivation (Sakly et al., 2013). Therefore, the intake of spineless cactus could be responsible for the increase in feed intake (Table 2), due to the suppression of hepatic glucose production (Viñoles et al., 2008) contributing to improve energy balance and minimize the BWL (Table 4) whose effect was reflected in a lower WEI (Fig. 4).

## Conclusion

The addition of 1% of spineless cactus (according to the live weight of the sows) to the diet of lactating sows reduces blood glucose levels during the first 2 weeks of lactation, generating increased voluntary feed intake and reduction in body weight loss in lactating sows, and its consequence is reflected in a reduction of the weaning-estrus interval.
